# Dasatinib induces DNA damage and activates DNA repair pathways leading to senescence in non-small cell lung cancer cell lines with kinase-inactivating *BRAF* mutations

**DOI:** 10.18632/oncotarget.6376

**Published:** 2015-11-23

**Authors:** Shaohua Peng, Banibrata Sen, Tuhina Mazumdar, Lauren A. Byers, Lixia Diao, Jing Wang, Pan Tong, Uma Giri, John V. Heymach, Humam N. Kadara, Faye M. Johnson

**Affiliations:** ^1^ Department of Thoracic/Head & Neck Medical Oncology, The University of Texas MD Anderson Cancer Center, Houston, Texas, USA; ^2^ AstraZeneca Pharmaceuticals, London, UK; ^3^ The University of Texas Graduate School of Biomedical Sciences, Houston, Texas, USA; ^4^ Department of Bioinformatics and Computational Biology, The University of Texas MD Anderson Cancer Center, Houston, Texas, USA; ^5^ Department of Translational Molecular Pathology, The University of Texas MD Anderson Cancer Center, Houston, Texas, USA

**Keywords:** lung cancer, BRAF, TAZ, DNA damage, kinase inhibition

## Abstract

Improved therapies are greatly needed for non-small cell lung cancer (NSCLC) that does not harbor targetable kinase mutations or translocations. We previously demonstrated that NSCLC cells that harbor kinase-inactivating *BRAF* mutations (^KI^*BRAF*) undergo senescence when treated with the multitargeted kinase inhibitor dasatinib. Similarly, treatment with dasatinib resulted in a profound and durable response in a patient with ^KI^*BRAF* NSCLC. However, no canonical pathways explain dasatinib-induced senescence in ^KI^*BRAF* NSCLC. To investigate the underlying mechanism, we used 2 approaches: gene expression and reverse phase protein arrays. Both approaches showed that DNA repair pathways were differentially modulated between ^KI^*BRAF* NSCLC cells and those with wild-type (WT) *BRAF*. Consistent with these findings, dasatinib induced DNA damage and activated DNA repair pathways leading to senescence only in the ^KI^*BRAF* cells. Moreover, dasatinib-induced senescence was dependent on Chk1 and p21, proteins known to mediate DNA damage-induced senescence. Dasatinib also led to a marked decrease in TAZ but not YAP protein levels. Overexpression of TAZ inhibited dasatinib-induced senescence. To investigate other vulnerabilities in ^KI^*BRAF* NSCLC cells, we compared the sensitivity of these cells with that of ^WT^*BRAF* NSCLC cells to 79 drugs and identified a pattern of sensitivity to EGFR and MEK inhibitors in the ^KI^*BRAF* cells. Clinically approved EGFR and MEK inhibitors, which are better tolerated than dasatinib, could be used to treat ^KI^*BRAF* NSCLC. Our novel finding that dasatinib induced DNA damage and subsequently activated DNA repair pathways leading to senescence in ^KI^*BRAF* NSCLC cells represents a unique vulnerability with potential clinical applications.

## INTRODUCTION

Non-small cell lung cancer (NSCLC) is a common and rapidly fatal cancer for which targeted therapies have been markedly effective in about 20% of patients, specifically those with *EGFR* mutations, *ROS1* rearrangements, or *EML4-ALK* translocations. However, only a minority of the remaining 80% of patients likely have targetable, activating kinase mutations or translocations, and there is a great need to identify additional effective therapies [1]. We previously identified a patient with stage IV NSCLC harboring a novel *BRAF* mutation (Y472C) that had a near complete radiographic response to the multitargeted kinase inhibitor dasatinib as the sole therapy; the patient lived without active cancer for 7 years following treatment [2]. We discovered that ^Y472C^*BRAF* is a kinase-inactivating *BRAF* mutation (^KI^*BRAF*) and that NSCLC cells that harbor ^KI^*BRAF* undergo senescence when exposed to dasatinib, whereas NSCLC with wild-type *BRAF* (^WT^*BRAF*) or kinase-activating mutations is resistant to dasatinib *in vitro* and in patients [3].

The RAS/RAF/MEK/ERK pathway plays an important role in the progression of many human cancers. Once activated by surface receptors, RAS recruits RAF, a serine/threonine kinase, to the cell membrane and activates it. RAF then phosphorylates MEK, which in turn phosphorylates and activates ERK, leading to cancer progression or senescence depending on the degree of ERK activation and crosstalk with other signaling pathways [4]. The 3 RAF proteins (A, B, and C) can form homodimers and heterodimers [5]. BRAF is by far the most frequently mutated isoform [6]. *BRAF* mutations can result in increased or decreased BRAF kinase activity, as well as kinase-neutral mutations, and *BRAF* mutations occur in 3–8% of patients with NSCLC [7–11] and many other tumor types [12]. ^KI^*BRAF* still paradoxically activates MEK/ERK to levels higher than those in cells with ^WT^*BRAF* via heterodimerization with CRAF (Raf-1) [13–16]. Similarly, inhibition of ^WT^*BRAF* or expression of ^KI^*BRAF* increases CRAF-BRAF binding, activates CRAF, and enhances MEK/ERK activation [3, 14–16].

The underlying mechanism of dasatinib-induced senescence in ^KI^*BRAF* NSCLC cells is obscure. Dasatinib inhibits the activity of Src and Abl, as well as nearly 40 distinct kinase targets [17, 18]. Dasatinib weakly inhibits BRAF, although only at concentrations higher than those needed to induce senescence, and it can induce BRAF-CRAF dimerization and CRAF activation in cells with activated RAS or ^KI^*BRAF* mutations [3, 19]. Although RAF dimerization was found to be necessary for dasatinib sensitivity, nilotinib, a kinase inhibitor with a similar kinase profile that also produced robust RAF dimerization, did not induce senescence. Another potent Src/Abl inhibitor, bosutinib, did not induce senescence [3]. Currently there are no well-defined, canonical pathways that explain the observed dasatinib-induced senescence in ^KI^*BRAF* NSCLC cells.

We sought to define the underlying mechanism leading to dasatinib-induced senescence in ^KI^*BRAF* NSCLC cells. We used 2 approaches: gene expression arrays and reverse phase protein array (RPPA), in which we simultaneously examined the expression of 137 proteins and phosphoproteins in ^KI^*BRAF* and ^WT^*BRAF* NSCLC cell lines at baseline and following dasatinib treatment. Our approach was limited by the existence of only 2 NSCLC cell lines with endogenous ^KI^*BRAF*, but nonetheless we determined that dasatinib induced DNA damage and subsequently activated DNA repair pathways and decreased TAZ expression leading to senescence in ^KI^*BRAF* NSCLC cells. TAZ is part of the Hippo pathway that is a complex network of at least 35 proteins that converge on a core kinase cassette that consists of MST1/2, LATS1/2, SAV1, and MOB [20]. LATS1/2 phosphorylates the transcriptional co-activators YAP and TAZ that results in their ubiquitin-mediated proteolysis. TAZ has recently been defined as a novel oncogene in NSCLC cells where TAZ knock-down results in decreased anchorage-independent growth *in vitro* and tumor growth *in vivo*; TAZ overexpression causes transformation of bronchial epithelial cells [21].

## RESULTS

### DNA repair pathways are differentially modulated between ^KI^*BRAF* and ^WT^*BRAF* NSCLC cells treated with dasatinib

**Figure 1:**
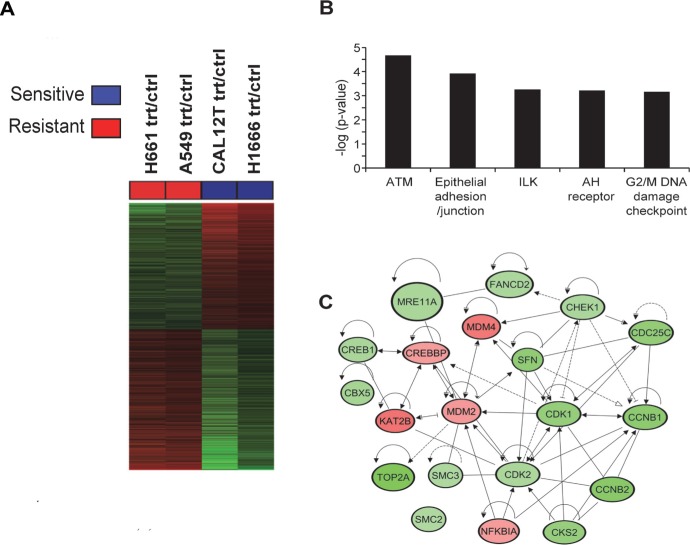
Comparison of gene expression changes following treatment with dasatinib in non-small cell lung cancer cells with and without kinase-inactivating BRAF mutations (^KI^BRAF) **A.** Heat map of the 2061 gene features that were differentially modulated by dasatinib in ^KI^*BRAF* cells compared to wild-type *BRAF* (^WT^*BRAF*; fold change ≥ 1.35, *P* < 0.05). Treatment versus control ratios (rows) for each of the differentially expressed gene features were derived and used for clustering gene features and cell lines (see Methods). Columns represent the cell lines and rows represent treatment/control ratios of the gene features (red, gene feature ratio up-regulated in sensitive compared to resistant cells; green, gene feature ratio down-regulated in sensitive cells). **B.** The five most significant (indicated by inverse log of *P*-value) canonical pathways, identified by pathways analysis using IPA, that were differentially modulated by dasatinib in the ^KI^*BRAF* compared to the ^WT^*BRAF* NSCLC cells. **C.** Differentially modulated gene features in the ATM and G2/M DNA damage checkpoint pathways were topologically organized using IPA to reveal functional gene-gene interactions; green, dasatinib modulated gene features down-regulated in ^KI^*BRAF* compared to ^WT^*BRAF* cells; red, dasatinib-modulated gene features that were up-regulated in ^KI^*BRAF* cells. Trt, treated with 150 nM dasatinib for 72 hours. Crtl, vehicle control.

We used gene expression arrays as an unbiased method to investigate mechanisms underlying dasatinib-induced senescence. We performed gene expression profiling of ^KI^*BRAF* NSCLC cells (H1666 and Cal12T, which harbor ^G466V^*BRAF*) and ^WT^*BRAF* NSCLC cells (A549, H661) that were incubated for 72 hours with 150nM dasatinib or vehicle control. We chose 72 hours because we previously showed that incubation for 72 hours was required to induce irreversible senescence [3]. Using the Affymetrix Human Genome U133 Plus 2.0 array platform, we identified profiles that were modulated by dasatinib in each of the sensitive (^KI^*BRAF* H1666 and Cal12T cells) and resistant (^WT^*BRAF* A549 and H661) groups. These profiles were then cross compared among the two cell line groups to determine differential effects of dasatinib in ^KI^*BRAF* relative to ^WT^*BRAF* cells (Figure 1A). We found 2061 gene features corresponding to 1458 genes that were differentially modulated by dasatinib between ^KI^*BRAF* and ^WT^*BRAF* cells (fold change ≥ 1.35, *P* < 0.05; Figure 1A).

We then performed functional pathways and gene set enrichment analyses using Ingenuity Pathways Analysis (IPA) to identify pathways and gene sets that were significantly differentially modulated by dasatinib between ^KI^*BRAF* and ^WT^*BRAF* cells (Figure 1B, Supplementary Table 1). This analysis demonstrated that the ATM and G2M/DNA damage checkpoint pathways were markedly differentially modulated between ^KI^*BRAF* and ^WT^*BRAF* cells, suggesting that dasatinib induced DNA damage in ^KI^*BRAF* cells (Figure 1B–1C).

Because E2F target genes mediate senescence, we specifically examined E2F target genes using gene set enrichment. This analysis revealed significantly suppressed (indicated by negative z-scores) function of E2F1, 2, and 3 in the ^KI^*BRAF* cell lines (Supplementary Figure 1, Supplementary Table 3). Moreover, the majority of the E2F1, 2 and 3 target genes were down-regulated in dasatinib-treated ^KI^*BRAF* cell lines relative to similarly treated ^WT^*BRAF* cell (Supplementary Table 2). Although dasatinib affected E2F signaling at early time points in both ^KI^*BRAF* and ^WT^*BRAF* cells, the downregulation was distinct between the two groups at 72 hours (Supplementary Figure 2). Of note, we also found, based on downstream gene expression, significantly suppressed function of *TP53* in the dasatinib-treated ^KI^*BRAF* cell lines compared with similarly treated ^WT^*BRAF* cell lines (Supplementary Table 2).

### DNA repair and TAZ are differentially modulated between ^KI^*BRAF* and ^WT^*BRAF* NSCLC cells treated with dasatinib

To further elucidate the mechanism underlying dasatinib-induced senescence in ^KI^*BRAF* NSCLC cells, we used RPPA as a second approach to simultaneously evaluate the expression of 137 proteins and phosphoproteins in ^KI^*BRAF* and ^WT^*BRAF* NSCLC cells incubated for 72 hours with dasatinib.

**Figure 2:**
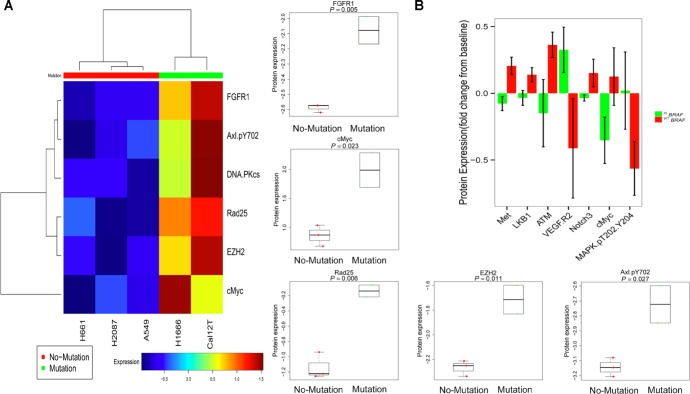
Proteins involved in DNA repair and TAZ are differentially expressed and modulated between non-small cell lung cancer cells with kinase-inactivating BRAF mutations (^KI^BRAF) and those with wild-type BRAF (^WT^BRAF) **A.** Basal protein expression of 137 proteins and phosphoproteins was compared between ^KI^*BRAF* and ^WT^*BRAF* cells. To generate the heat map, we used Pearson correlation distance between proteins and Euclidean distance between samples. We used the Ward method for both genes' and the samples' linkage rule. A 2-sample *t* test was applied and 6 markers were identified at a false discovery rate of 0.45. **B.** The mean protein expression of all 137 measured proteins before and after 72 hours of incubation with 150 nM dasatinib was compared between ^KI^*BRAF* and ^WT^*BRAF* cells. Error bars represent standard deviation. *P* < 0.05 for all 7 proteins shown.

Six proteins were differentially expressed at baseline (*P* < 0.05) between ^KI^*BRAF* and ^WT^*BRAF* cells, including 2 proteins involved with DNA repair: DNA-dependent protein kinase, catalytic subunit (DNA-PKcs) and ERCC3 (Rad25; Figure 2A). We compared the modulation of all proteins after 72 hours of incubation with dasatinib between ^KI^*BRAF* and ^WT^*BRAF* cells. Although only 7 proteins were significantly differentially modulated (*P* < 0.05; Figure 2B), we considered this RPPA a discovery set and subsequently examined many proteins that were differentially regulated between the 2 groups (*P* > 0.05, Supplementary Figure 3).

**Figure 3:**
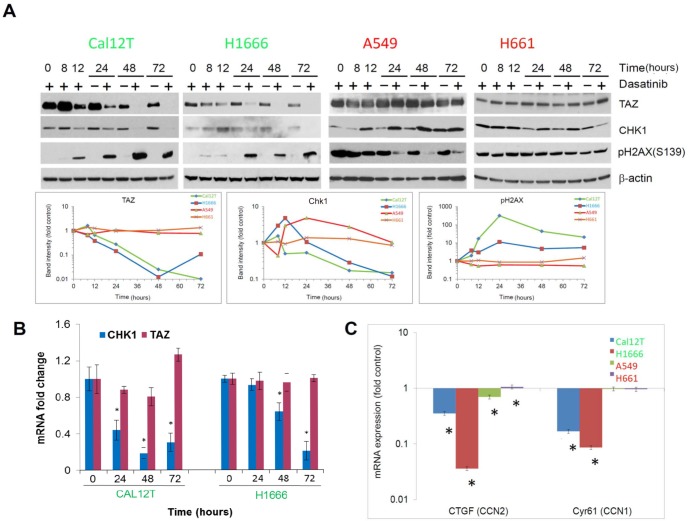
Chk1 and TAZ are differentially modulated between non-small cell lung cancer cells with BRAF mutations (^KI^BRAF) and those with wild-type BRAF (^WT^BRAF) **A.** Western blot analysis showing changes in protein expression for cells incubated with 150nM dasatinib for the indicated times. Bands were quantitated in the line graphs at the bottom. **B.** mRNA levels of Chk1 and TAZ were measured using quantitative polymerase chain reaction for the indicated times following incubation with 150nM dasatinib. **C.** mRNA levels of TAZ target genes were measured using quantitative polymerase chain reaction for the indicated times following incubation with 150nM dasatinib. Error bars represent standard deviation. *P < 0.05.

To confirm the results of the RPPA and to examine expression over time, we incubated NSCLC cells with dasatinib for various durations up to 72 hours and performed Western blot analysis of various proteins. For the longer incubation periods (i.e., 24 hours or more), we included vehicle-treated samples to control for signaling changes due to cell density (Figure 3A). Dasatinib induced a sustained increase in levels of the phosphorylated H2A histone family, member X (pH2AX, γH2AX) and decreases in levels of Chk1 and TAZ protein expression in ^KI^*BRAF* cells but not in ^WT^*BRAF* cells. The phosphorylation of γH2AX at serine 139 is an early response to double-strand DNA breaks [22].

H2AX is phosphorylated by several phosphoinositide-3-kinase related protein kinases (PIKKs) including ATM, ATR, and DNA-PK [22]. Both ATM and DNA-PK were phosphorylated in ^KI^*BRAF* cells treated with dasatinib but at time points later than the phosphorylation of γH2AX suggesting an alternative mechanism or a lack of sensitivity in the phospho-specific antibodies (Supplementary Figure 4).

To investigate the Hippo pathway further, we measured TAZ mRNA levels, which did not change after treatment with dasatinib in ^KI^*BRAF* cells (Figure 3B), demonstrating post-transcriptional regulation. Additionally, pLats1/2 was increased in ^KI^*BRAF* cells after dasatinib treatment (Supplementary Figure 3). Lats1/2 phosphorylates TAZ resulting in its ubiquitin-mediated proteolysis. Although the increase in pLats1/2 was modest, the TAZ protein has a very short half-life [23] and may be sensitive to this small change. To confirm the effect of dasatinib on TAZ, we measured the expression of 2 TAZ transcription targets using quantitative polymerase chain reaction following incubation with 150nM dasatinib for 72 hours and found that CCN1 and CCN2 were significantly decreased only in ^KI^*BRAF* cells (Figure 3C). Likewise, using the gene expression array data, we found that the majority of TAZ-regulated genes were downregulated in the ^KI^*BRAF* cell lines treated with dasatinib (Supplementary Figure 5).

### Dasatinib induces DNA damage in ^KI^*BRAF* NSCLC cells

**Figure 4:**
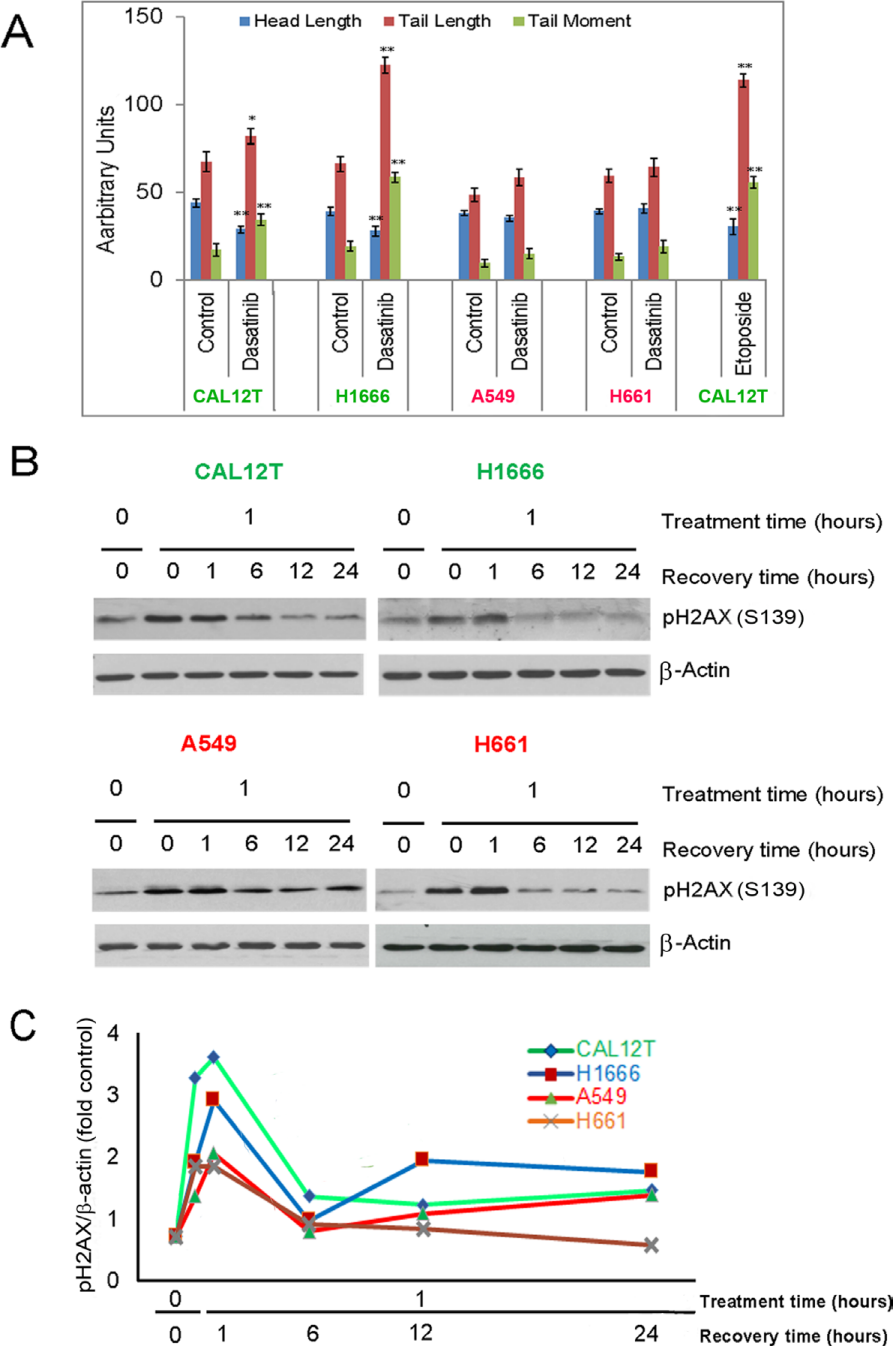
Dasatinib induces DNA damage in non-small cell lung cancer cells with kinase-inactivating BRAF mutations (^KI^BRAF) **A.** ^KI^*BRAF* cells (Cal12T, H1666) and wild-type *BRAF* cells (H661, A549) were incubated with 150nM dasatinib for 72 hours and DNA damage was measured using the COMET assay. COMET-Assay IV software was used to estimate the tail length, head length, and tail moment. Error bars represent standard deviation. *P < 0.05 compared with control. **B.** NSCLC cells were incubated with 5 μM etoposide for 1 hour and then medium was changed to remove drug. Resolution of phosphorylated γH2AX was measured using Western blotting (representative blot shown) with quantitation of γH2AX bands normalized for β-actin expression **C.**

The differential effects of dasatinib on several DNA repair proteins led us to examine the effects of dasatinib on DNA damage. We employed the COMET/single-cell gel electrophoresis assay, which nonspecifically measures both double-strand and single-strand DNA breaks by separating fragmented DNA from intact DNA using electrophoresis. Results of this assay demonstrated significant dasatinib-induced DNA damage in ^KI^*BRAF* but not in ^WT^*BRAF* cells. The average tail length and tail moment increased only in ^KI^*BRAF* cells (Figure 4A).

To determine if ^WT^*BRAF* and ^KI^*BRAF* cell lines had different abilities to repair DNA, we treated cells with the DNA damaging agent etoposide and measured resolution of phosphorylated H2AX. Both the ^WT^*BRAF* and ^KI^*BRAF* cell lines recovered fully within 6 hours (Figure 4B–4C).

### Knockdown of p21 or overexpression of Chk1 and TAZ protects ^KI^*BRAF* NSCLC cells from dasatinib-induced senescence

**Figure 5:**
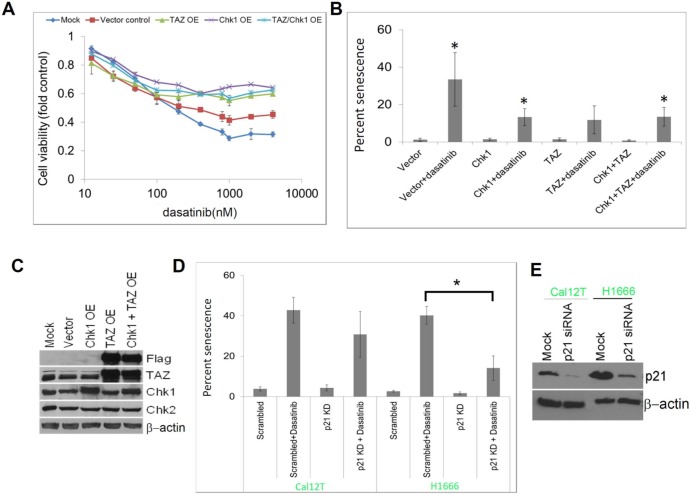
Dasatinib-induced senescence is mediated by Chk1, TAZ, and p21 **A.** H1666 cells were transfected with DNA vectors containing Chk1, TAZ, or both and sensitivity to dasatinib was measured using the MTT assay at the indicated drug concentrations after 72 hours of incubation. **B.** Senescence was estimated using β-galactosidase staining in transfected cells treated with 150nM dasatinib or vehicle control for 72 hours. **C.** Overexpression (OE) was confirmed by Western blot analysis. **D.** Non-small cell lung cancer cell lines with kinase-inactivating *BRAF* mutations (Cal12T, H1666) were transfected with siRNA targeting p21 or a scrambled control and incubated with 150nM dasatinib or vehicle control for 72 hours. Senescence was estimated using β-galactosidase staining. **E.** Knockdown (KD) was confirmed by Western blot analysis. Error bars represent standard deviation. *P < 0.05 compared with control.

To determine whether the downregulation of Chk1 or TAZ was functionally significant, we overexpressed Chk1, TAZ, or both in H1666 cells and measured sensitivity to dasatinib (Figure 5A). We found that overexpression of either TAZ or Chk1 increased dasatinib resistance and decreased dasatinib-induced senescence (Figure 5B), although overexpression of either protein did not affect expression of the other (Figure 5C). In contrast, Lats2 knockdown, which was insufficient to affect TAZ expression, did not alter dasatinib sensitivity (Supplementary Figure 6).

Senescence that is triggered by DNA damage is characterized by p21 accumulation. To determine whether dasatinib-induced senescence is mediated by p21, we knocked down p21 with siRNA. We have previously observed p21 accumulations in Cal12T and H1666 cells treated with dasatinib. In the current study, p21 knockdown led to decreased dasatinib-induced senescence (Figure 5D–5E).

### Dasatinib does not lead to oncogene-induced senescence

**Figure 6:**
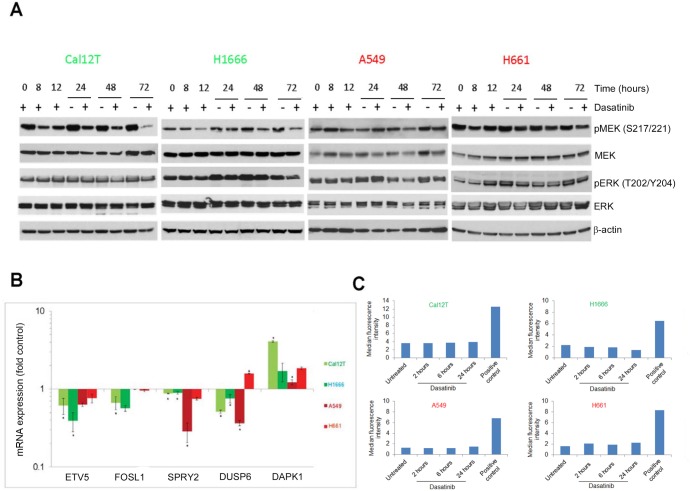
Dasatinib does not induce oncogene-induced senescence Non-small cell lung cancer cell lines with kinase-inactivating *BRAF* mutations (Cal12T, H1666) or wild-type *BRAF* (H661, A549) were incubated with 150nM dasatinib for the indicated times (A, C) or for 72 hours (B) Activation of the MEK/ERK pathway was measured using Western blot analysis with the indicated antibodies **A.** or quantitative polymerase chain reaction for downstream transcriptional targets **B.** Error bars represent standard deviation. *P < 0.05 compared with control. **C.** The production of reactive oxygen species was estimated using a fluorogenic probe that exhibits fluorescence upon oxidation by reactive oxygen species.

The sensitivity of ^KI^*BRAF* cells NSCLC to MEK inhibitors (below) and the fact that activation of oncogenes, including *BRAF*, paradoxically leads to senescence that depends on the modulation of MEK/ERK signaling [24, 25] led us to examine ERK and MEK activation following treatment with dasatinib in ^KI^*BRAF* NSCLC cells (H1666, Cal12T) and ^WT^*BRAF* NSCLC cells (A549, H661) using phosphospecific antibodies (Figure 6A). We observed no sustained changes caused by dasatinib in the ^KI^*BRAF* cells. The transient activation of ERK in A549 cells that we observed may be due to dasatinib-induced dimerization, which was previously described [3, 19].

Owing to feedback inhibition, including activation of downstream phosphatases, phosphorylated ERK and MEK may not be the ideal measurement of MEK/ERK activation. We also measured ERK signaling output using a subset of ERK-dependent genes that consisted of 5 downstream genes (Figure 6B) [26]. One disadvantage of this method is that other pathways can also influence the expression of these 5 genes. However, as in the Western blot analysis, we observed no consistent changes caused by dasatinib that were unique in the ^KI^*BRAF* cells. Additionally, dasatinib did not lead to the production of reactive oxygen species (ROS) that mediate oncogene-induced senescence (Figure 6C).

### Overexpression of ^WT^*BRAF* reduces dasatinib-induced senescence and DNA damage in ^KI^*BRAF* NSCLC cells

In our previous work, we transfected NSCLC cell lines that have endogenous ^WT^*BRAF* with two different ^KI^*BRAF* constructs and found increased sensitivity to dasatinib [3]. Likewise we found that transfection of ^wt^*BRAF* into Cal12T partially rescued the phenotype with reduced dasatinib induced-apoptosis and DNA damage (Supplementary Figure 7). Full rescue was not expected as ^KI^*BRAF* is not an inactive molecule despite a lack of kinase activity. ^KI^*BRAF* paradoxically activates MEK/ERK to levels higher than those in cells with ^WT^*BRAF* via heterodimerization with CRAF (Raf-1) [13–16].

### ^KI^*BRAF* NSCLC cells are sensitive to inhibitors of EGFR and MEK but not SRC, ABL, or RAF

**Figure 7:**
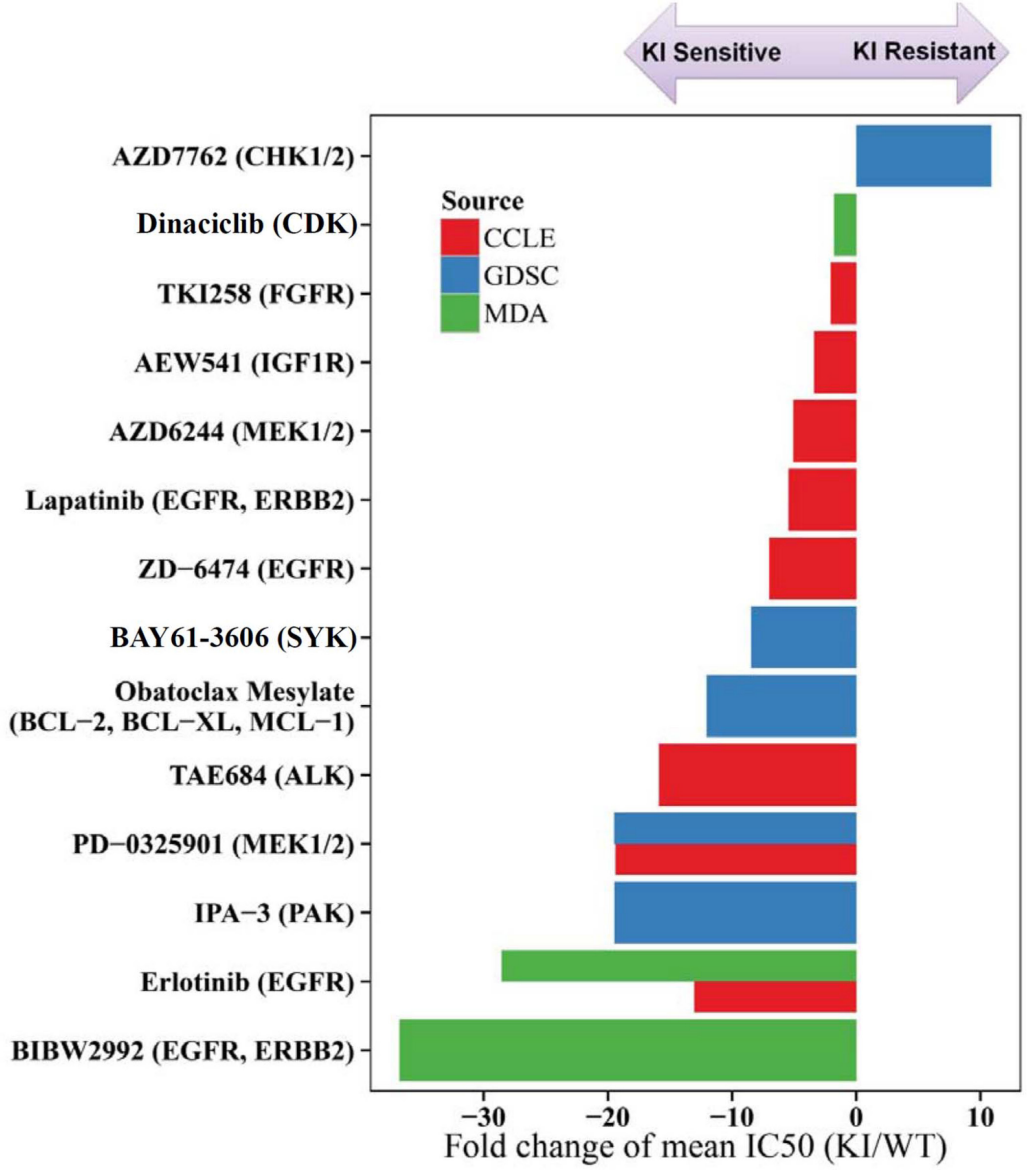
Non-small cell lung cancer (NSCLC) cells with kinase-inactivating (KI) BRAF mutations are more sensitive to inhibitors of EGFR and MEK than NSCLC cells with wild-type (WT) BRAF We compared the sensitivity of NSCLC cell lines with kinase-inactivating *BRAF* mutations (Cal12T, H1666) to those with wild-type *BRAF* using 79 drugs (Supplementary Table 5). A 2-sample *t* test was used to determine whether the mean half-maximal inhibitory concentration (IC_50_; after log_10_ transformation) was statistically different between the 2 groups. Only drugs with significantly different sensitivities between the groups (*P* < 0.05) are shown. Drug sensitivity data were obtained from 2 publically available databases (Genomics of Drug Sensitivity in Cancer [GDSC] and Cancer Cell Line Encyclopedia [CCLE]) and from 13 drugs tested at MD Anderson (MDA).

Another approach we employed to identify pathways of vulnerability in ^KI^*BRAF* cells was to examine sensitivity to drugs with a broad range of targets. To achieve this goal, we downloaded data from 2 large, publically available cell line screening databases (GDSC and CCLE - described below) and tested 13 compounds to identify drugs that were particularly effective in ^KI^*BRAF* cells compared with ^WT^*BRAF* cells [27, 28]. In the Genomics of Drug Sensitivity in Cancer (GDSC) database, 57 drugs were tested in both Cal12T and H1666 cells, along with 65–70 ^WT^*BRAF* NSCLC cell lines. In the Cancer Cell Line Encyclopedia (CCLE) database, 13 drugs were tested in both Cal12T and H1666 cells, along with 86–89 ^WT^*BRAF* NSCLC cell lines. We tested 13 drugs with a broad spectrum of targets in 45 NSCLC cell lines (Cal12T and H1666 and various ^WT^*BRAF* cell lines) using the Cell Titer Glo assay. All tested drugs are listed in Supplementary Tables 4 and 5. Among all 79 unique drugs (4 drugs overlapped), we identified 14 drugs with sensitivities that were statistically different between ^KI^*BRAF* and ^WT^*BRAF* NSCLC cells (Figure 7). ^KI^*BRAF* cells were consistently sensitive to inhibitors of the ErbB family and MEK1/2. Consistent with our previously published data, Cal12T and H1666 were not particularly sensitive to inhibition of SRC or ABL, which are both potently inhibited by dasatinib [3].

To determine if drugs that were effective in ^KI^*BRAF* NSCLC cells functioned similarly to dasatinib, we treated cells with BIBW2992 and PD0325901 and measured γH2AX, senescence, and apoptosis. These drugs did not result in significant senescence (< 10%) or γH2AX expression but did cause apoptosis demonstrating that the combination worked through a distinct mechanism from dasatinib in ^KI^*BRAF* NSCLC cells (Supplementary Figure 8).

The selective effects of dasatinib on NSCLC cells harboring ^KI^*BRAF* suggests that the combination of BRAF inhibitors with dasatinib would be effective in ^WT^*BRAF* NSCLC which would have a broader clinical application. In our previous publication, we combined vemurafenib with dasatinib and demonstrated synergy in NSCLC cell lines with ^wt^*BRAF* [3]. To further characterize the underlying mechanism for this synergy, we incubated NSCLC cells with ^WT^*BRAF* and measured apoptosis, senescence, TAZ and γH2AX. As with the EGFR and MEK inhibitors, the combination did not function similarly to dasatinib in ^KI^*BRAF* NSCLC cells. The combination led to apoptosis in A549 cells and senescence in H661 cells; none had modulated TAZ, CHK1, or γH2AX levels (Supplementary Figure 9).

## DISCUSSION

In the current study, we investigated the mechanism leading to dasatinib-induced senescence in ^KI^*BRAF* NSCLC cells and discovered that dasatinib leads to DNA damage, changes in DNA repair signaling pathways, and the downregulation of TAZ expression in these cells. Dasatinib-induced senescence was dependent on p21 accumulation and the loss of TAZ and Chk1. Overexpression of either TAZ or Chk1 did not affect expression of the other protein, suggesting that these were independent events.

Senescence can be induced by telomere shortening, DNA damage, or oncogene activation. The DNA damage response is initiated when the MRN (MRE11-RAD50-NBs1) complex is recruited to areas of double-strand breaks. This complex recruits ATM, which phosphorylates H2AX (γH2AX) and Chk1. The DNA damage response may in fact function in most cases of senescence because telomere shortening leads to DNA damage that induces the DNA damage response. Replication stress can also perturb heterochromatin leading to the formation of senescence associated heterochromatic foci [29] that we observed following dasatinib treatment in NSCLC cells with ^KI^*BRAF* [3]. Oncogene-induced senescence can lead to the production of ROS and replication stress leading to the DDR [30]. Dasatinib did not induce ROS production in any of the cell lines nor did we observe sustained ERK/MEK activation, suggesting that the NSCLC cell lines did not undergo oncogene-induce senescence.

Although dasatinib alone has never been shown to lead to DNA damage prior to this study, it has been shown to do so in combination with other agents. Inhibition of c-ABL by dasatinib delays DNA repair following radiation-induced DNA damage in leukemia and head and neck cancer cells [31, 32]. The combination of dasatinb and Herceptin in HER2 expressing breast cancer cell lines leads to DNA damage although neither agent alone did so [33]. One unanswered question is the mechanism by which dasatinib leads to DNA damage selectively in ^KI^*BRAF* NSCLC cell lines. One potential mechanism is through CRAF. We previously demonstrated that dasatinib indirectly inhibits CRAF only in cells with ^KI^*BRAF* but not ^WT^*BRAF*. Additionally, CRAF knock down affected only those NSCLC with ^KI^*BRAF* [3]. Although we did not demonstrate a differential effect on MEK/ERK signaling based on *BRAF* mutation, CRAF can signal independently of MEK/ERK [34]. In support of this idea, expression of ^KI^*BRAF* (*D594A*) in mice, but not kinase active ^V600E^*BRAF*, leads to CRAF-dependent aneuploidy that is not MEK dependent [35]. Additional evidence for the role of RAF in dasatinib-induced senescence is that we previously demonstrated that transfection of ^KI^*BRAF* into NSCLC cell lines increased their sentivity to dasatinib but when an additional mutation was added that prevented BRAF dimerization, no sensitization was observed [3]. MEK-independent CRAF signaling via MST1/2 is also a potential link between dasatinib-induced RAF dimerization, Chk1 and the Hippo pathway. The Hippo pathway is a complex network of at least 35 proteins, including MST1/2, that converge on a core kinase cassette leading to phosphorylation of Lats1/2 [20]. CRAF can bind to MST1/2, the mammalian orthologue of Hippo, and interfere with its dimerization and activation [36, 37]. CRAF and the RAS-association domain family 1 isoform A (RASSF1A) compete for binding to MST1/2. RASSF1A binds to and stabilizes MST1/2 leading to activation of the HIPPO pathway [38]. ATM, Aurora A and Chk1 can phosphorylate RASSF1A leading to its activation [39, 40]. Lats1/2 phosphorylates the transcriptional co-activators YAP and TAZ that results in their ubiquitin-mediated proteolysis. YAP is a stable protein but TAZ is very unstable with a half-life of less than 2 hours [23]. TAZ has recently been defined as a novel oncogene in NSCLC cells where TAZ knock-down results in decreased anchorage-independent growth *in vitro* and tumor growth *in vivo*; TAZ overexpression causes transformation of bronchial epithelial cells [21]. In dasatinib-treated ^KI^*BRAF* NSCLC cell lines, YAP expression was not affected and phosphorylated Lats1/2 increased slightly whereas levels of TAZ were profoundly decreased. These findings, along with a lack of concurrent decreases in TAZ mRNA demonstrate that dasatinib affects post transcriptional control of TAZ in ^KI^*BRAF* NSCLC cells. Additionally, we were not able to detect RASSIF1A expression in our NSCLC cell lines with any commercially available antibodies (data not shown).

We and others previously found that cells with ^KI^*BRAF* mutations still activate MEK and may be particularly sensitive to its inhibition [3, 15]. Indeed we discovered that H1666 and Cal12T cell lines were more sensitive to MEK as well as upstream receptor tyrosine kinases – particularly EGFR than NSCLC cell lines lacking ^KI^*BRAF* mutations. These increased drug sensitivities were found independently from 3 different screens and with 4 distinct EGFR inhibitors and 2 distinct MEK inhibitors supporting a pathway-specific effect rather than an off target drug effect.

We used two approaches to determine that dasatinb led to DNA damage and senescence selectively in NSCLC cell lines with ^KI^*BRAF* mutations. This work builds on our prior studies demonstrating a marked and durable effect of dasatinb in a NSCLC patient with a ^KI^*BRAF* mutation [2] and dasatinib-induced senescence that was dependent upon RAF heterodimerization in NSCLC cell lines [3]. These results have potential clinical application as there is a striking need to discover targeted therapies for cancers that lack driver, activating kinase mutations and because dasatinib, EGFR inhibitors and MEK inhibitors are all approved drugs.

## MATERIALS AND METHODS

### Materials

Dasatinib, BIBW2992, PD0325091 and Etoposide were purchased from Selleck Chemicals (Houston, TX) and prepared as 20 mM stock solutions in DMSO. Antibodies used included anti-YAP-TAZ, pLats, Lats1, Lats2, pMST1/2, pH2AX, MEK, pMEK(S217/221), ERK, pERK(T202/Y204), pRb, pS6, LC3A/B, ATM, pChk1, Chk1, pChk2, Chk2, pCDC25c, pATRIP, p27, p21, pp38, β-catenin, cyclin D1, survivin, p4EBP1, pSMAD2(S465–467), p70S6K, pCDC2, CDC6, γH2AX, ATR, pDNA-PK(S2056) and DNA-PK, (Cell Signaling Technology, Danvers, MA); BRAF and cyclin E (Santa Cruz, Dallas, TX) and ATM, Flag M2 and β-actin (Sigma-Aldrich, St. Louis, MO); pATM(S1981) (GeneTex, Irvine, CA). Predesigned siRNAs of the target genes were purchased from Dharmacon (Pittsburgh, PA) or Thermo Scientific (Rodckford, IL). pcDNA4-Chk1-Flag and pCMV5-TOPO-3Xflag-TAZ plasmids were purchased from Addgene (Cambridge, MA). ^WT^*BRAF* plasmid was provided by Dr. W. Kolch (Systems Biology Irland and The Conway Institute, University College Dublin).

### Cell culture

Human NSCLC cell lines were obtained from Dr. John Heymach, confirmed with DNA fingerprinting, and maintained as previously described [41]. Cal12T was purchased from DSMZ (Braunschweig, Germany).

### Western blot analysis

Western blot analysis was performed as previously described [42]. Briefly, cells were lysed on ice and the lysates were centrifuged at 20,000*g* for 5 minutes at 4°C and then boiled with 1× Laemmli sample buffer for 5 minutes. Equal amounts of protein aliquots were resolved with SDS–polyacrylamide gel electrophoresis, transferred to nitrocellulose membranes, immunoblotted with a primary antibody, and detected with a horseradish peroxidase–conjugated secondary antibody (Bio-Rad Laboratories, Hercules, CA) and ECL reagent (Pierce Biotech, Rockford, IL).

### Quantitative polymerase chain reaction (PCR)

Total RNA was isolated from NSCLC cell lines treated as indicated in the figure legends using an RNeasy mini kit (Qiagen, Valencia, CA) according to the manufacturer's instructions. Total RNA (2 μg) was converted into cDNA using 1× Moloney murine leukemia virus buffer, 1 μL RNasin, 10 μmol/L random hexamer, 500 μmol/L deoxyribonucleotide triphosphates, 100 mg/mL bovine serum albumin, and 1.5 μL Moloney murine leukemia virus reverse transcriptase enzyme in a final reaction volume of 20 μL. The reaction mixture was incubated at 42°C for 2 hours, and the reaction was terminated by heating the mixture at 99°C for 5 minutes and then at 5°C for 5 minutes as described previously [43].

The mRNA levels for the indicated genes were measured with SYBR green–based real-time PCR in triplicate as described previously [43]. The primers were designed using Primer-BLAST (National Center for Biotechnology Information; Supplementary Table 6). Each cDNA sample was amplified using SYBR Green PCR Master Mix (Applied Biosystems, Grand Island, NY) according to the manufacturer's protocol. The PCR products were detected and their dissociation curves were calculated using the ABI Prism 7500 fast real-time PCR system (Applied Biosystems, Foster City, CA). The level of the housekeeping ribosomal gene L32 (*Rpl32*) was used as an internal control. Individual data sets were normalized with control vehicle-treated cells; DDC_T_ values were normalized with L32 as an internal control.

### COMET assay

The alkaline COMET/single-cell gel electrophoresis assay was performed according to the manufacturer's suggested protocol (OxiSelect COMET assay Kit, Cell Biolabs Inc., San Diego, CA). In brief, OxiSelect COMET slides were coated with COMET agarose mixed with experimental cells (10^4^ cells/mL). After on-slide cell lysis with lysis buffer, cells were subjected to electrophoresis for 30 minutes under 1 volt/cm at 4°C. After electrophoresis, cells were stained with Vista Green DNA dye and visualized with epifluorescence microscopy using a FITC filter [44, 45]. Tail length, head length, and tail moment were measured using COMET-Assay IV software V4.3 (Perceptive Instruments, Bury St Edmunds, UK).

### Overexpression and knockdown of TAZ, Lats1, p21, Chk1 and ^WT^*BRAF*

To knock down expression, we used predesigned sets of 4 independent siRNA sequences of the target genes (siGENOME SMARTpool, Dharmacon, and Thermo Scientific, Pittsburgh, PA). To achieve overexpression of Chk1 and TAZ, we used pcDNA4-Chk1-Flag for Chk1 and pCMV5-TOPO-3Xflag-TAZ for TAZ (Addgene). Controls included cells that were mock transfected (no siRNA or DNA), those transfected with vector alone (for overexpression experiments), and those transfected with a nontargeting (scrambled) siRNA (for knockdown experiments). Cells were harvested, washed, and suspended (2 × 10^6^/100 μL) in Nucleofector V solution (Lonza Group, Walkersville, MD). siRNA (200 pmol/100 μL), DNA (3 μg/100 μL), or controls were added and electroporated using the U031 or U024 Nucleofector program (Lonza) as described previously [46]. ^WT^*BRAF* overexpression was performed by Lipofectamine 3000 Transfection Kit (ThermoFisher Scientific, Grand Island, NY) according to the manufacturer's instruction. Overexpression or knockdown was confirmed using Western blot analysis as described above.

### Reverse-phase protein array proteomic analysis

Antibody validation and RPPA analysis were performed as described previously [47]. Briefly, protein lysate was collected from NSCLC cell cultures after 72 hours in 150nM dasatinib or vehicle control. Protein lysates (1 μg/μL) were printed (2470 Arrayer; Aushon Biosystems, Burlington, MA) on nitrocellulose-coated slides (Grace Bio-Labs, Bend, OR) and immunostaining was performed using a DakoCytomation-catalyzed system and diaminobenzidine colorimetric reaction. Spot intensities were quantified using MicroVigene software (VigeneTech Inc., Carlisle, MA).

### Cell viability assays

Cell viability was measured using the MTT assay as previously described [42]. In the indicated cell lines, viability was assessed using the CellTiter Glo proliferation assay (Promega, Madison, WI) according to the manufacturer's specifications. For both assays, 6 replicates were tested at each concentration, and each test was completed at least twice on different days.

### Senescence-associated β-galactosidase staining and apoptosis assay

Senescence-associated β-galactosidase staining was performed using the β-galactosidase staining kit (Cell Signaling Technology) according to the manufacturer's suggested protocol and visualized under an Olympus 1X71 phase microscope (Melville, NY). In brief, cells were fixed with formaldehyde at room temperature for 10 minutes followed by overnight staining with X-galactoside at 37°C [3]. Fields that contained at least 100 cells were counted in triplicate. To measure apoptosis, cells were treated with the drugs as described in the figure legends and digested by Accutase Cell Detachment Solution (BD Biosciences, San Jose, CA), stained by using FITC Annextin V Apoptosis Detection Kit (BD Biosciences, San Jose, CA). Apoptotic cells was detected by Gallios Flow Cytometer (Beckman Coulter, Inc., Brea CA) and analyzed by Kaluza software in MD Anderson Cancer Center flow cytometry core lab.

### Gene expression

NSCLC cells were incubated with vehicle control or 150nM dasatinib for 72 hours and total RNA was isolated using the RNeasy Mini kit (Qiagen). The quality of the RNA was measured using an Agilent 2100 Bio-analyser (Agilent Technologies, Palo Alto, CA). RNA samples were processed for microarray profiling using the GeneChip Human Genome U133 Plus 2.0 platform (Affymetrix, Santa Clara, CA). *In vitro* transcription, cDNA target preparation, cDNA fragmentation and labelling, hybridization, and chip scanning for subsequent generation of raw data files (.CEL files) were performed by Asuragen, Inc (Austin, TX) as previously described [48]. Microarray expression data were analysed using BRB-ArrayTools version 4.3 developed by Richard Simon and The BRB-ArrayTools development team [49]. Raw data were quantified using background correction and normalized using Robust Multichip Analysis in the R environment [50]. Basic quality control was assessed using graphical summaries of array intensities and Bland-Altman (M-versus-A) plots. Minimum information about microarray experiments–compliant data were submitted to the Gene Expression Omnibus under series GSE69395. Two 2-sample *t* tests with random variance were performed in sensitive and resistant cells separately to identify gene features in either cell line group that were significantly (absolute fold-change ≥ 1.35, *P* < 0.05) differentially expressed following treatment with dasatinib [51]. Ratios of the differentially expressed gene features (*n* = 2,061) in treated cells compared with control cells were then derived, and these ratios were cross-compared between dasatinib-sensitive and -resistant cells. Pathway analysis of the differentially expressed gene features was performed using Ingenuity pathway analysis.

### Drug sensitivity

Half-maximal inhibitory concentration (IC_50_) data from the Cancer Cell Line Encyclopedia database were obtained from http://www.broadinstitute.org/ccle/home [27]. IC_50_ data from the Genomics of Drug Sensitivity in Cancer database were downloaded from http://www.cancerrxgene.org/ [52]. IC_50_ values from our data were estimated using the drexplorer package as previously described [53]. IC_50_ values were then determined from the best dose-response model identified by residual standard error. The concordance correlation coefficient was used to assess reproducibility from biological replicates. A 2-sample *t* test was used to determine whether mean IC_50_ values (after log_10_ transformation) were statistically different (*P* < 0.05) between ^WT^*BRAF* and ^KI^*BRAF* cells.

### Reactive oxygen species (ROS)

ROS was measured by cellROX oxidative stress reagent (Life Technologies, Carlsbad, CA) according to the manufacturer's instructions. In brief, cells were treated with 150nM dasatinib for the indicated times and then incubated with cellROX green fluorogenic probe, which exhibits strong fluorogenic signals upon oxidation. Cells were then washed and analyzed using flow cytometry. For a positive control, each cell line was treated with Tert-butyl hydroperoxide to produce ROS. SYTOX red dead cell stain was used to exclude dead cells during flow cytometry analysis.

## SUPPLEMENTARY FIGURES AND TABLES














