# Supplementary material for: Dasatinib induces DNA damage and activates DNA repair pathways leading to senescence in non-small cell lung cancer cell lines with kinase-inactivating BRAF mutations
Source: Oncotarget. 2015 Nov 23;7(1):565–79. doi: 10.18632/oncotarget.6376 (PMC4808018; doi:10.18632/oncotarget.6376)
Supplement: Supplementary file 1 [file oncotarget-07-0565-s001.pdf]

SUPPLEMENTARY FIGURES AND TABLES

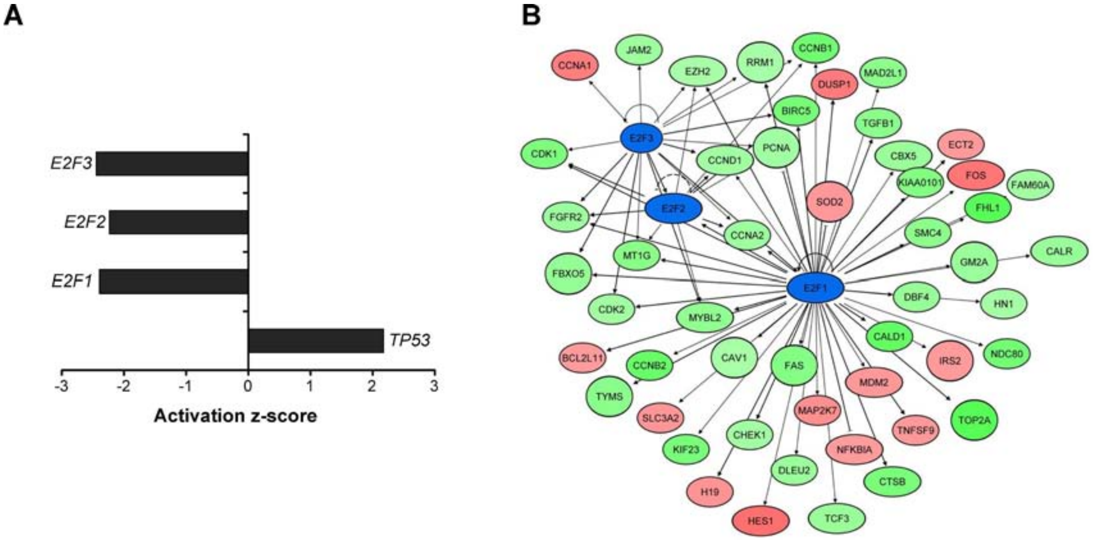

Supplementary Figure S1: A. Gene set enrichment analysis demonstrated that E2F1, 2, 3 and TP53 function and downstream signaling is suppressed between dasatnib-treated non-small cell lung cancer cells with kinase-inactivating *BRAF* mutations (^{KI}*BRAF*) compared to similarly treated cells with wild-type *BRAF* (^{WT}*BRAF*). A negative activation z score represents down-regulated function in ^{KI}*BRAF* compared to ^{WT}*BRAF* cells. **B.** Differentially modulated target genes downstream of E2F1, 2 and 3 were topologically organized using IPA to reveal functional gene-gene interactions; green, dasatinib modulated gene features down-regulated in ^{KI}*BRAF* compared to ^{WT}*BRAF* cells; red, dasatinib-modulated gene features that were up-regulated in ^{KI}*BRAF* cells.

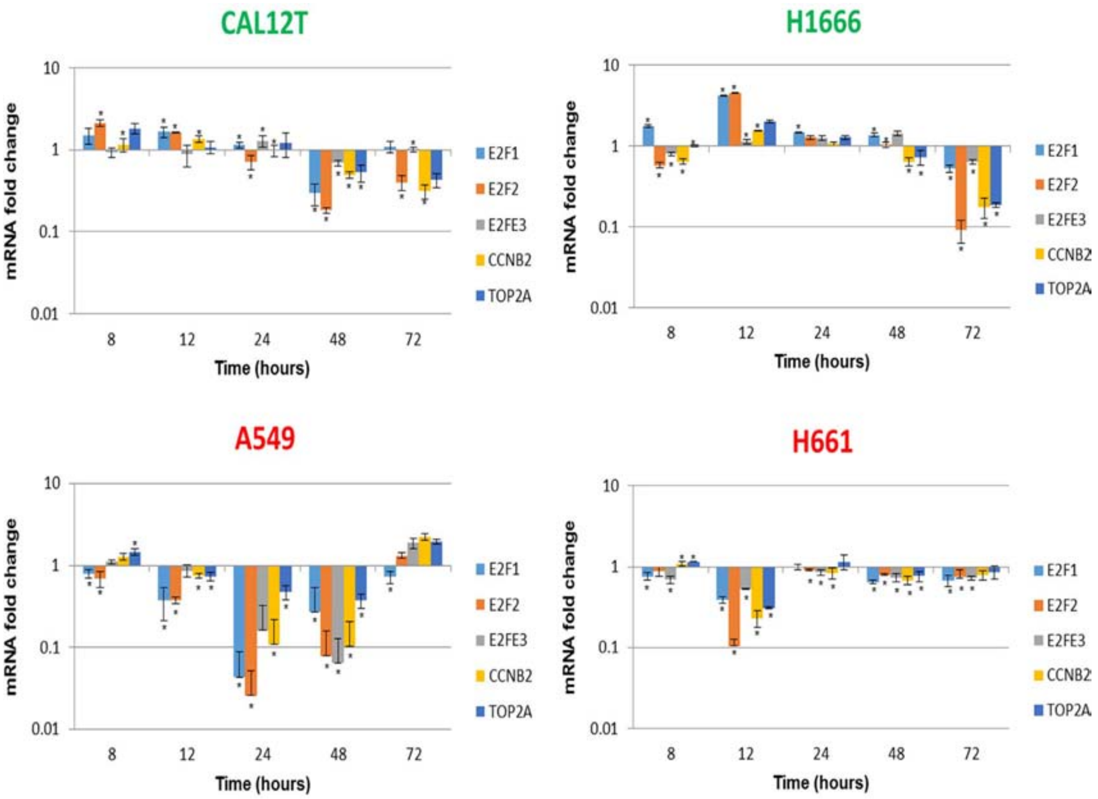

Supplementary Figure S2: E2F and target genes were differentially modulated following 72 hours of dasatinib treatment in NSCLC cell lines with [KI]*BRAF* compared to cells with [WT]*BRAF*. NSCLC cell lines were incubated with 150nM dasatinib for the indicated times and qPCR was performed to measure levels of the indicated genes. *$P < 0.05$ compared with control.

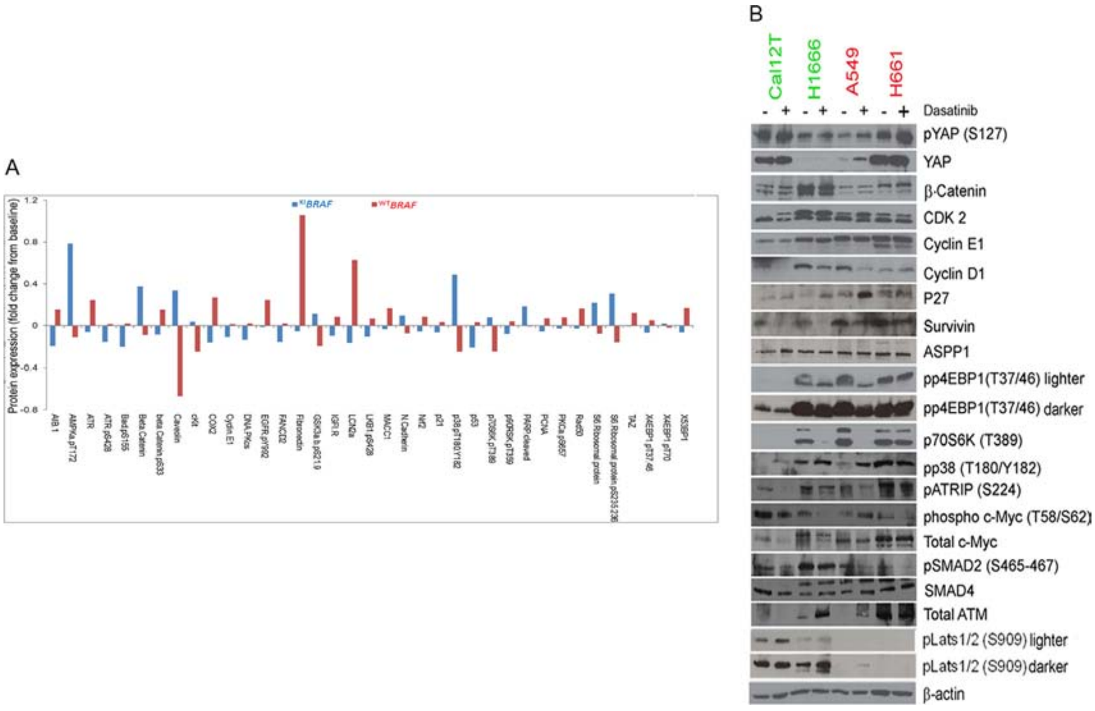

Supplementary Figure S3: A. Expression of all 137 measured proteins before and after 72 hours of incubation with 150nM dasatinib was compared between non-small cell lung cancer cells with kinase-inactivating *BRAF* mutations (^{KI}*BRAF*) and cells with wild-type *BRAF* (^{WT}*BRAF*). Proteins that were differentially regulated between ^{KI}*BRAF* cells and ^{WT}*BRAF* cells were included in the figure. None of these differences reached statistical significance. **(B)** Western blot analysis of levels of indicated proteins in non-small cell lung cancer cells incubated with 150nM dasatinib or vehicle control for 72 hours.

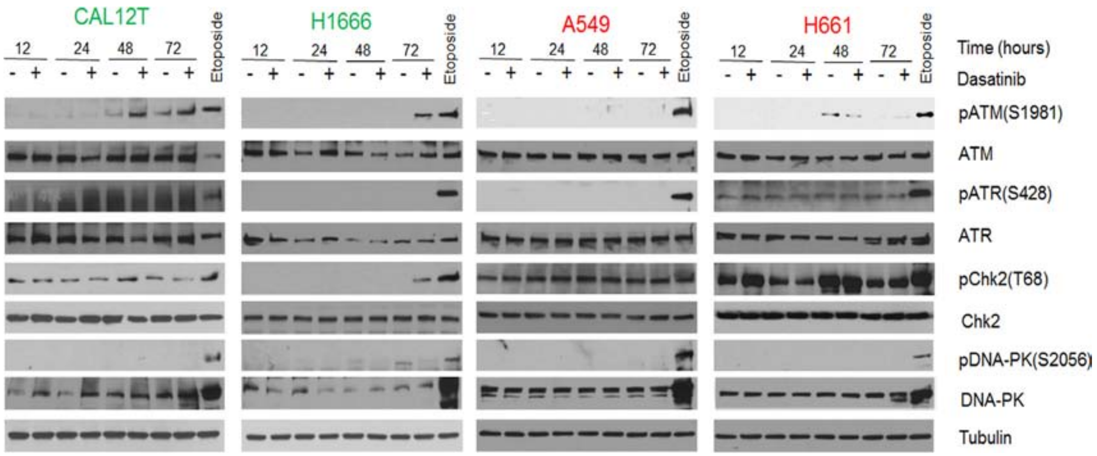

Supplementary Figure S4: DNA damage response proteins in ^{KI}*BRAF* and ^{WT}*BRAF* NSCLC cell lines. Western blot analysis showing changes in protein expression for cells incubated with 150nM dasatinib for the indicated times. 5 µM etoposide was used as a positive control.

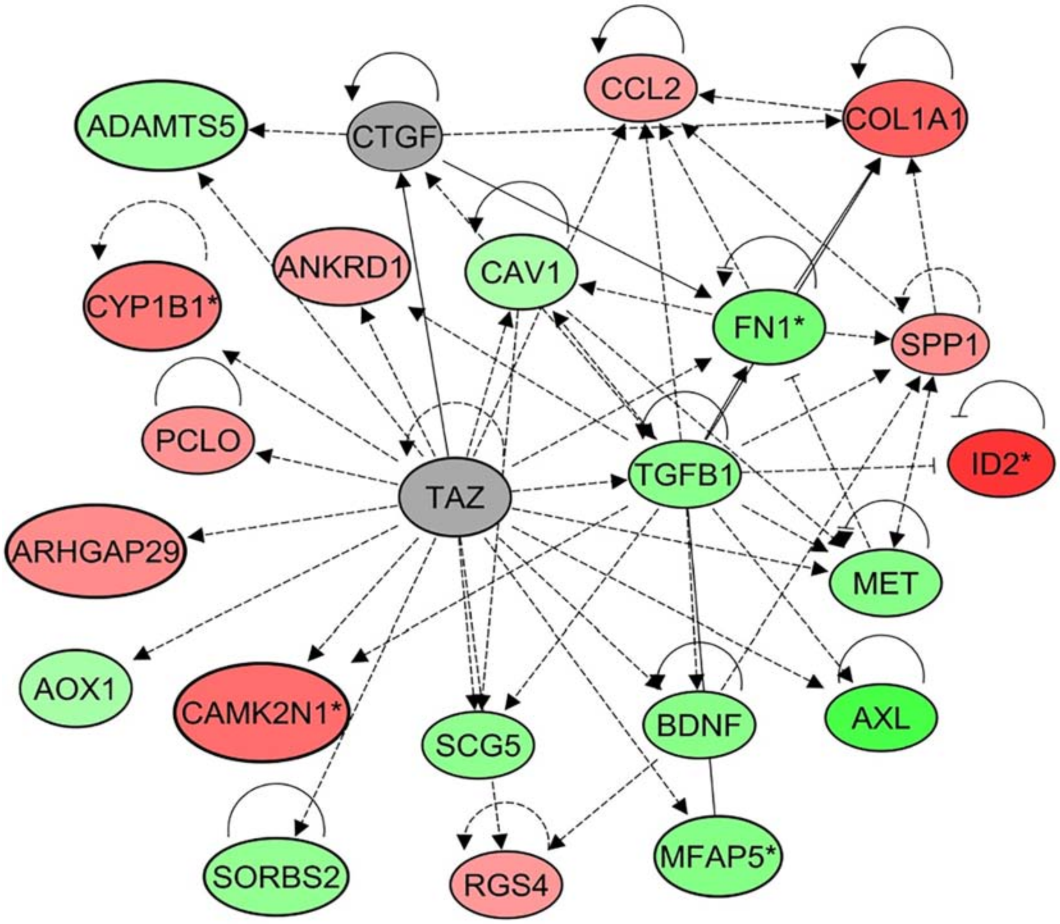

Supplementary Figure S5: Differentially modulated TAZ target genes were topologically organized using IPA.

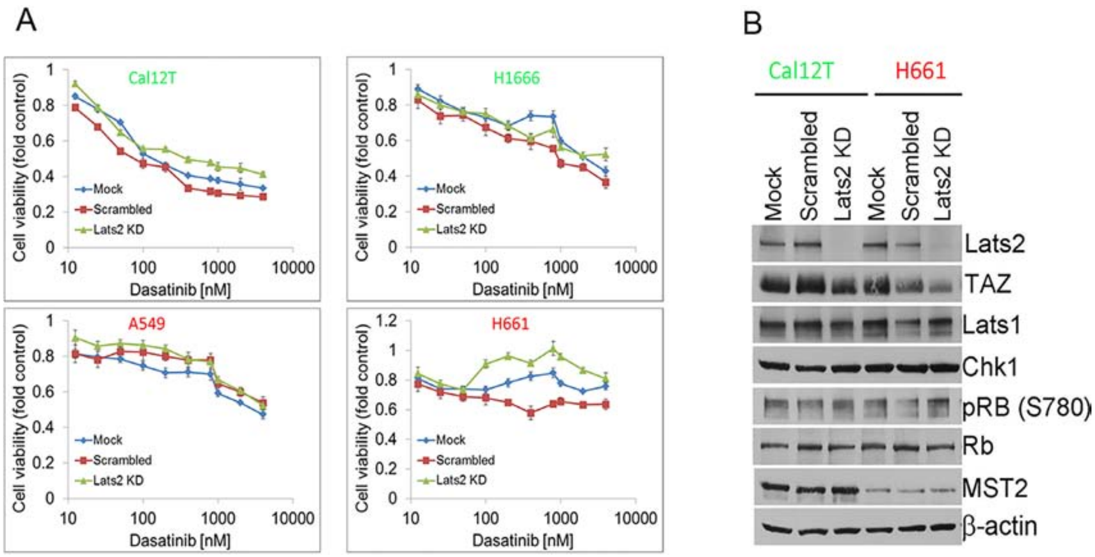

Supplementary Figure S6: A. H1666 cells were transfected with siRNA against Lats2 and sensitivity to dasatinib was measured using the MTT assay at the indicated drug concentrations after 72 hours of incubation. (B) Knockdown (KD) was confirmed by Western blot analysis.

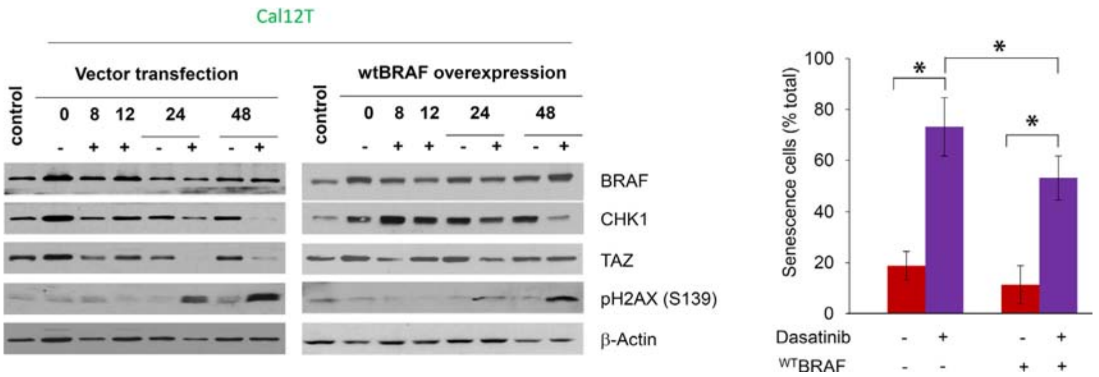

Supplementary Figure S7: Overexpression of ^{WT}*BRAF* in NSCLC cells with ^{KI}*BRAF* reduced dasatinib-induced senescence, DNA damage, and effect on TAZ and Chk1 expression. Cal12T cells were transfected with a DNA vector containing BRAF or vector alone and Western blot analysis performed with the indicated antibodies at the indicated times **(B)** Senescence was estimated using β-galactosidase staining in transfected cells treated with 150nM dasatinib or vehicle control for 72 hours. *$P < 0.05$ compared with control or as indicated.

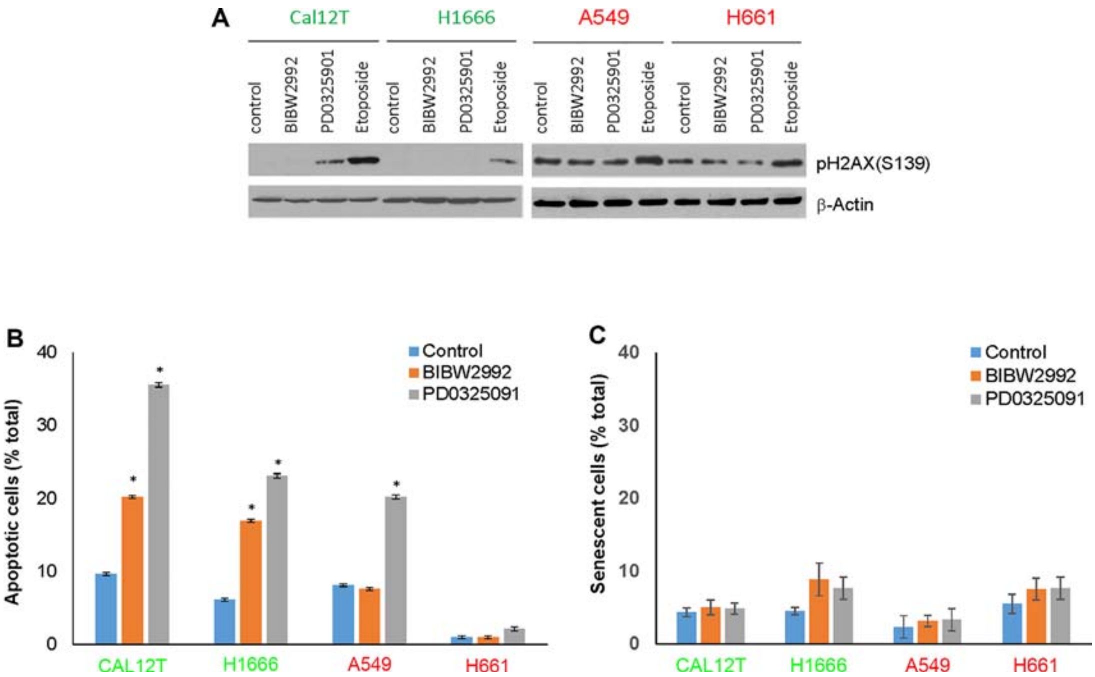

Supplementary Figure S8: The biological effects of the EGFR and MEK inhibitors BIBW2992 and PD0325901 are distinct from those of dasatinib in NSCLC with ^{KI}***BRAF*.** NSCLC cell lines were incubated with 150nM BIBW2992 or 1 µM PD0325901 for 72 hours. **A.** Western blot analysis performed with the indicated antibodies. 5 µM etoposide was used as a positive control for γH2AX expression. **B.** Apoptosis was estimated using Annexin V staining. **C.** Senescence was estimated using β-galactosidase staining. Error bars represent standard deviation. *P < 0.05 compared with control.

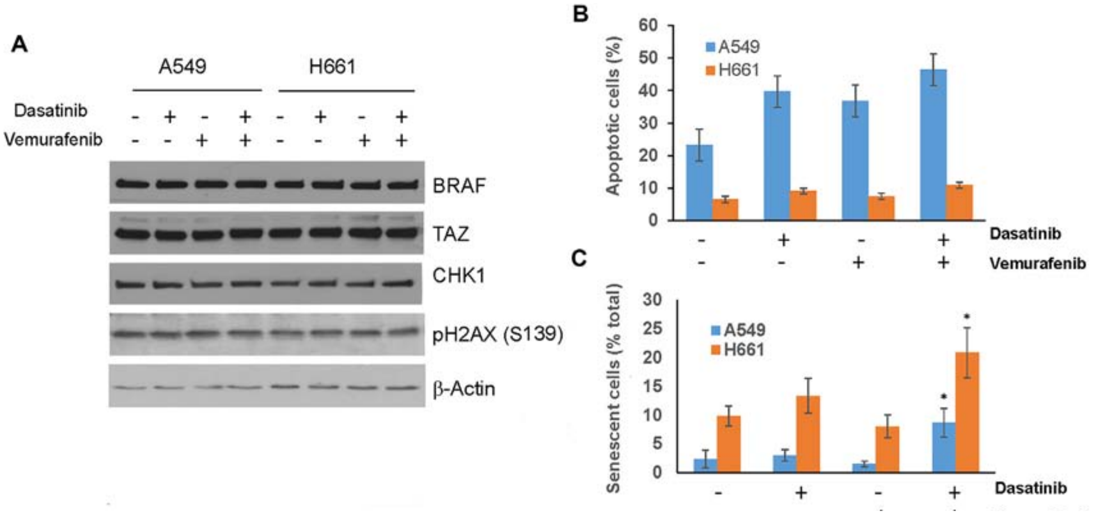

Supplementary Figure S9: The biological effects of the combination BRAF inhibition with dasatinib in NSCLC with [WT]*BRAF* are distinct from those of dasatinib in NSCLC with [KI]*BRAF*. NSCLC cell lines were incubated with 2 μM vemurafenib or 150nM dasatinib for 72 hours. **A.** Western blot analysis performed with the indicated antibodies. **B.** Apoptosis was estimated using Annexin V staining **C.** Senescence was estimated using β-galactosidase staining. Error bars represent standard deviation. *$P < 0.05$ compared with control.

Supplementary Table 1: Pathways and gene sets that were significantly differentially modulated by dasatinib between ^{KI}*BRAF* and ^{WT}*BRAF* cells

Supplementary Table 2: Differential modulation by dasatinib between ^{KI}*BRAF* and ^{WT}*BRAF* cells of E2F1, 2, and 3 target genes

Supplementary Table 3: Differential modulation by dasatinib between ^{KI}*BRAF* and ^{WT}*BRAF* cells of E2F1, 2, and 3 and TP53

Supplementary Table 4: List of drugs tested in NSCLC cell lines in the Genomics of Drug Sensitivity in Cancer, Cancer Cell Line Encyclopedia, and at MD Anderson listed separately

Supplementary Table 5: Compiled list of drugs tested in NSCLC cell lines in the Genomics of Drug Sensitivity in Cancer, Cancer Cell Line Encyclopedia, and at MD Anderson

Supplementary Table 6: Primers used for SYBR green–based real-time PCR
